# Ticks on the move—climate change-induced range shifts of three tick species in Europe: current and future habitat suitability for *Ixodes ricinus* in comparison with *Dermacentor reticulatus* and *Dermacentor marginatus*

**DOI:** 10.1007/s00436-022-07556-x

**Published:** 2022-06-01

**Authors:** Sarah Cunze, Gustav Glock, Judith Kochmann, Sven Klimpel

**Affiliations:** 1 Institute of Ecology, Evolution and Diversity, Goethe-University, Max-von-Laue-Str. 13, 60438 Frankfurt/Main, Germany; 2 Senckenberg Gesellschaft Für Naturforschung, Senckenberg Biodiversity and Climate Research Centre, Senckenberganlage 25, 60325 Frankfurt/Main, Germany

**Keywords:** Ecological niche modelling, Species distribution modelling, Range expansion

## Abstract

Tick-borne diseases are a major health problem worldwide and could become even more important in Europe in the future. Due to changing climatic conditions, ticks are assumed to be able to expand their ranges in Europe towards higher latitudes and altitudes, which could result in an increased occurrence of tick-borne diseases.

There is a great interest to identify potential (new) areas of distribution of vector species in order to assess the future infection risk with vector-borne diseases, improve surveillance, to develop more targeted monitoring program, and, if required, control measures.

Based on an ecological niche modelling approach we project the climatic suitability for the three tick species *Ixodes ricinus*, *Dermacentor reticulatus* and *Dermacentor marginatus* under current and future climatic conditions in Europe. These common tick species also feed on humans and livestock and are vector competent for a number of pathogens.

For niche modelling, we used a comprehensive occurrence data set based on several databases and publications and six bioclimatic variables in a maximum entropy approach. For projections, we used the most recent IPCC data on current and future climatic conditions including four different scenarios of socio-economic developments.

Our models clearly support the assumption that the three tick species will benefit from climate change with projected range expansions towards north-eastern Europe and wide areas in central Europe with projected potential co-occurrence.

A higher tick biodiversity and locally higher abundances might increase the risk of tick-borne diseases, although other factors such as pathogen prevalence and host abundances are also important.

**Supplementary information:**

The online version contains supplementary material available at 10.1007/s00436-022-07556-x.

## Introduction

Climate change is generally considered one of the main drivers of biodiversity loss (Pereira et al. 2010; Román-Palacios and Wiens 2020). However, some species are able to expand their range or increase their abundance locally and thus benefit from changing climatic conditions. A particular interest exists in assessing the potential changes in habitat suitability due to climate change for medically or economically relevant species. Thermophilic species (in Europe, e.g., species with focal distribution in Southern Europe) are assumed to benefit from increasing temperatures (Cunze et al. 2016a; Ju et al. 2015; Steger et al. 2020).

As blood-sucking ectoparasites, ticks are vectors for a variety of pathogens, including protozoa, viruses, bacteria and nematodes (Sonenshine and Roe 2014), which are considered causative agents of a number of diseases. In Europe, Lyme borreliosis (LB) and tick-borne encephalitis (TBE) are the most common tick-borne diseases in humans (Süss 2011; Wormser et al. 2006), with *Ixodes ricinus* being the main vector species.

Tick-borne diseases have been considered a major health problem in recent decades, and it has been argued that, by affecting vector biology and disease transmission, climate change could have a positive effect on the occurrence of tick-borne diseases (Gray et al. 2009). On the one hand, ticks as vectors could benefit from climate change and thus increase their abundance locally, expand their range or extend their activity phase during the year due to mild winters (Dautel et al. 2008; Lindgren et al. 2000). On the other hand, vector competency and replication rates of pathogens could also be affected by rising temperatures (Ostfeld and Brunner 2015). Climate change can alter human behavior, which is seen as a very important driver of tick-borne diseases (Whitehorn and Yacoub 2019). In addition, host availability could impact pathogen prevalence and transmission (Cagnacci et al. 2012). According to Heyman et al. (2010) and Medlock et al. (2013), host availability (abundance and diversity) is a crucial factor in terms of pathogen prevalence in tick populations; high biodiversity and a high population density of the hosts are suggested to decrease pathogen prevalence in the tick population by reducing co-feeding of many ticks together on the same host and thus, the risk of infection. This mechanism is due not only to the reduction in co-feeding, but also to the diversion of tick-host interactions to incompetent reservoir hosts. Thus, when the population density of hosts with low reservoir competence is high, a dilution effect may occur. Host community composition or species evenness play also an important role (Ogden and Tsao 2009).

*Ixodes ricinus* is the most abundant and widespread tick in Europe (Gray et al. 2009). The species is of major human health relevance as it is vector competent for a number of pathogens, including *Borrelia burgd*orferi sensu lato, tick-borne encephalitis virus, *Anaplasma phagocytophilum*, *Francisella tularensis*, *Rickettsia helvetica* and *R. monacensis*, *Babesia divergens* and *B. microti*, and *Neoehrlichia mikurensis*, Louping ill virus and Tribec virus (Medlock et al. 2013). In Europe, *Ixodes ricinus* covers a wide geographic range including Scandinavia, the British Isles, Central Europe, France, Spain, Italy, the Balkans, and Eastern Europe (ECDC 2020b). In recent decades, a range expansion of *I. ricinus* to higher latitudes as well as to higher altitudes, where it was previously considered absent, has been observed in Europe (Jaenson et al. 2012; Medlock et al. 2013). In addition, increases in their local abundances have been observed (Tomkins et al. 2014). These processes have been attributed to ongoing changes in climatic conditions (Gray et al. 2009; Lindgren et al. 2000; Medlock et al. 2013). But non-climatic factors such as landscape and anthropogenic drivers also matter (Medlock et al. 2013). It is assumed that a climate change-induced range expansion of this important vector species could also lead to an increase in associated tick-borne disease cases in Europe.

Besides *I. ricinus*, there are other vector competent tick species whose distributional patterns in Europe are potentially affected by climate change, among them *Dermacentor reticulatus* and *D. marginatus*. *Dermacentor reticulatus* is the second most reported tick species in Central Europe after *I.* ricinus (Rubel et al. 2016). *Dermacentor reticulatus* and *D. marginatus* mainly occur in southern parts of Europe, but due to climate change, conditions could also become increasingly suitable for these species in Central Europe. Both *Dermacentor* species are vector competent for various pathogens causing human and animal diseases and are thus of public interest (Rubel et al. 2016). *Rickettsia* species have been found in both species, especially *R. raoultii* and *R. slovaca* (Parola and Raoult 2001), causing tick-borne lymphadenopathy, a diseases that has been first reported in 1979 in France (Raoult et al. 1997). Since then, the disease has also been detected in Germany and is expected to spread to other European regions (Rieg et al. 2011).

The ranges of *D. reticulatus* and *D. marginatus* broadly overlap, but *D. reticulatus* is not reported from the Mediterranean area, but occurs further north in Europe than *D. marginatus* (Rubel et al. 2016). This could indicate that *D. marginatus* is less cold tolerant than *D. reticulatus*. Of the three tick species considered here, *I. ricinus* shows the widest distribution range in Europe.

As for many ectothermic arthropods, temperature and relative humidity are considered the principal limiting factors shaping the geographic range of the species (Tomkins et al. 2014), especially during off-host periods, which comprise over 90% of their lifetimes. Whereas low winter temperatures can be the principal limiting factor for the geographic distribution of the tick species (Lindgren et al. 2000), it is also important how and where the species pass the winter. Adult stages of the three tick species considered here, are known to be exophilic. But the early stages of *Dermacentor* ticks are rarely or not at all found on vegetation which could indicate an endophilic behavior of these stages, i.e., nymphs remain within their host’s nest or burrow between blood meals (Immler 1973; Pfäffle et al. 2013), where they are less exposed to extreme climatic conditions. At higher temperatures, water balance becomes important. High temperatures in combination with low humidity cause ticks to visit microhabitats and pause their questing behaviour (Tomkins et al. 2014). Thus, high temperatures in combination with dryness in summer may limit ticks in their distribution range. Overall, specific requirements exist for different life-stages (egg, larva, nymph, adult) and a stable tick population requires that suitable conditions for all stages are met.

In this study, ecological niche models were generated for *I. ricinus*, *D. reticulatus* and *D. marginatus*, respectively, to project the climatic habitat suitability for these three tick species in Europe under current and future climate conditions. The three species are widespread in Europe but differ slightly in their ranges, which may at least be partly attributed to different abiotic and biotic requirements, i.e., different ecological niches. We hypothesise that *Ixodes ricinus*, showing the broadest distribution range of the considered species in Europe, should have a correspondingly broad niche. Besides, more thermophilic species are expected to be more strongly promoted by climate warming, thus, *Dermacentor marginatus* should show a temperature niche shifted towards an optimum at temperatures higher than for *D. reticulatus*. However, projected climate scenarios are complex and multifaceted.

## Material and methods

We projected the habitat suitability for the three tick species under current and future climatic conditions based on the ecological niche modelling approach. Ecological niche models are broadly applied in the fields of biology, nature conservation, and biogeography (Elith et al. 2011) and correlate data on species’ distribution to the environmental conditions prevailing in the study area. We here use the maximum entropy approach implemented in the software Maxent (Phillips et al.). Maxent is an often used and well performing modelling approach (Elith et al. 2011; Merow et al. 2013) that is able to produce robust results, even if the initial data is sparsely or irregularly sampled (Elith et al. 2006; Kramer-Schadt et al. 2013). As a presence-background approach, Maxent is particularly appropriate to model the habitat suitability in a dynamic situation when species’ ranges are subject to change, e.g., due to ongoing climatic changes, and when absence data are not reliable.

### Occurrence data

We have compiled occurrence data from several databases and publications (Estrada-Peña and La Fuente 2016; GBIF 2020; Rubel et al. 2016) accounting for records from the year 1970 onwards (Table S1 in the Supplementary Material for numbers of raw data for the three species). We then checked for common spatial and temporal errors applying the CoordinateCleaner R package (Zizka et al. 2019) and removed occurrence records that were identified as potentially incorrect. The remaining occurrence data (with 3365 records for *I. ricinus*, 904 records for *D. reticulatus*, and 1258 records for *D. marginatus*) were adapted to the grid of the environmental variables used for modelling (at a spatial resolution of 2.5 arc minutes), considering one occurrence records per grid cell at maximum of the training data set. This was done using the function rasterize in the R package raster (Hijmans and van Etten 2012). Thus, duplicates and closely located occurrence records were removed, resulting in a final data set containing 2803 occurrences for *I. ricinus*, 866 occurrences for *D. reticulatus*, and 1121 occurrences for *D. marginatus*. The distributions of final occurrence data are shown in Figure S1 in the Supplementary Material.

### Environmental data

We used data on climatic conditions provided by worldclim (version 2.0, Fick and Hijmans 2017). Nineteen so-called bioclimatic variables are available referring to the climatic conditions (monthly temperature and precipitation) empirically recorded over a period of 30 years from 1970 to 2000. These data were downloaded at a spatial resolution of 2.5 arc minutes and cropped to the extent of 32° north to 72° north and 11° west to 50° east.

From the 19 bioclimatic variables, we chose a subset of six ecologically relevant variables that are only slightly correlated among one another. For this purpose, we calculated the Pearson correlation coefficients r_P_ for each pair of the 19 bioclim variables using the function cor of R’s stats package (R Core Team 2021) and clustered them in a dendrogram based on the dissimilarity measure 1-|r_P_| using the R function hclust (method: complete linkage, Figure S2 in the Supplementary Material). We then excluded bio08, bio09, bio18 and bio19, which combine temperature and precipitation information in one layer. These variables show spatial discontinuities that are presumably not existent in the environmental conditions and should therefore be interpreted as artefacts (Escobar et al. 2014; Ruiz Barlett et al. 2019). With a threshold of 0.3 for the dissimilarity, which is the most commonly applied threshold to reduce collinearity in the environmental data sets (Dormann et al. 2013), we built groups of interrelated variables. We chose one representative of each group, which is considered ecologically relevant and easy to interpret. Specifically we chose: bio04 (temperature seasonality), bio05 (maximum temperature of warmest month), bio06 (minimum temperature of the coldest month), bio12 (annual precipitation), bio14 (precipitation of the driest month), and bio15 (precipitation seasonality).

We have only included climatic variables as predictor variables in ecological niche modelling, as climatic conditions are considered the most important drivers of species distribution patterns on a continental scale (Peterson 2011). As ectothermic arthropods, tick species are sensitive to climatic conditions, especially to temperature. Humidity and saturation are considered particularly important factors for the occurrence of ticks but are more relevant on a finer spatial scale (e.g., regional scale), as is vegetation cover.

For projections under future climatic conditions, we used the downscaled future climate projections according to the CNRM-ESM2-1 model (Seferian 2019) provided by worldclim (www.wordclim.org) for four Shared Socio-economic Pathways (SSPs): 126, 245, 370 and 585 for the time periods 2021–2040, 2041–2060, 2061–2080, and 2081–2100.

### Maxent

We used the default settings with some modifications; we only used linear, quadratic and product features and excluded hinge features (Cunze and Tackenberg 2015; Merow et al. 2013) and enhanced the number of maximum iterations to 50,000 to ensure convergence. For each species, we ran ten replicates using cross-validation and used their averages as the final models.

Maxent generates continuous values between zero and one for each pixel of the study area, which represent the modelled climatic suitability. These results are shown in habitat suitability maps for each species under current and future climatic conditions (Figures S3, S4, S5 in the Supplementary material), with warmer colors representing areas with higher climatic suitability. We used the 10% omission rate thresholds (Liu et al. 2005) to transform the logistic model output into binary maps: with suitable conditions in areas with modelling results above the threshold and unsuitable conditions in areas with modelling results below the threshold (Figures S6, S7, S8 in the Supplementary Material).

To evaluate model performance, we considered the AUC (area under the curve). The AUC value is an often used, threshold-independent measure, based on the receiver operating characteristic curve (ROC), which relates sensitivity (true positive rate) versus 1-specificity (true negative rate) (Liu et al. 2005). A greater AUC value (AUC ranges between 0 and 1) indicates a higher predictive model performance. Since the AUC value depends on the prevalence, it cannot be used to compare models of different species. We provide the mean AUC-value of the 10 replicates and the standard deviation.

### Further analysis

In order to evaluate if, to what extent, and where extrapolations can occur when projecting future habitat suitability (Elith et al. 2010), we used the multivariate environmental similarity surface (MESS) method implemented in Maxent (Figure S9 in the Supplementary Material). The MESS analysis helps to identify areas with future climatic conditions outside the range covered by the climatic conditions used for model training (near current conditions of 1970–2000). Modelling results for projected habitat suitability under future conditions outside the training range (extrapolations) should be treated with strong caution.

**Fig. 1:**
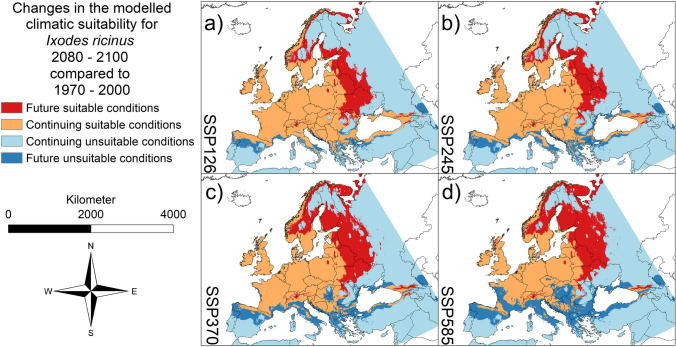
Projected future changes for *Ixodes ricinus* until 2081–2100. **a** SSP 126. **b** SSP 245. **c** SSP 370. **d** SSP 585. In dark blue: area projected as suitable under current climatic conditions but unsuitable under future climatic conditions (i.e., potential extinction). In light blue: area projected as unsuitable under current climatic conditions as well as under future climatic conditions (i.e., stable absence). In orange: area projected as suitable under current climatic conditions as well as under future climatic conditions (i.e., stable range). In red: area projected as unsuitable under current climatic conditions but suitable under future climatic conditions (i.e., potential new range). AUC = 0.7917 (average over 10 replicates using cross-validation, standard deviation = 0.000641959). Threshold to transform the logistic model output: 0.3368 (10% omission rate threshold). Maps were built using ESRI ArcGIS (Release 10.7, www.esri.com). Projection: Europe Albers Equal Area Conic

**Fig. 2:**
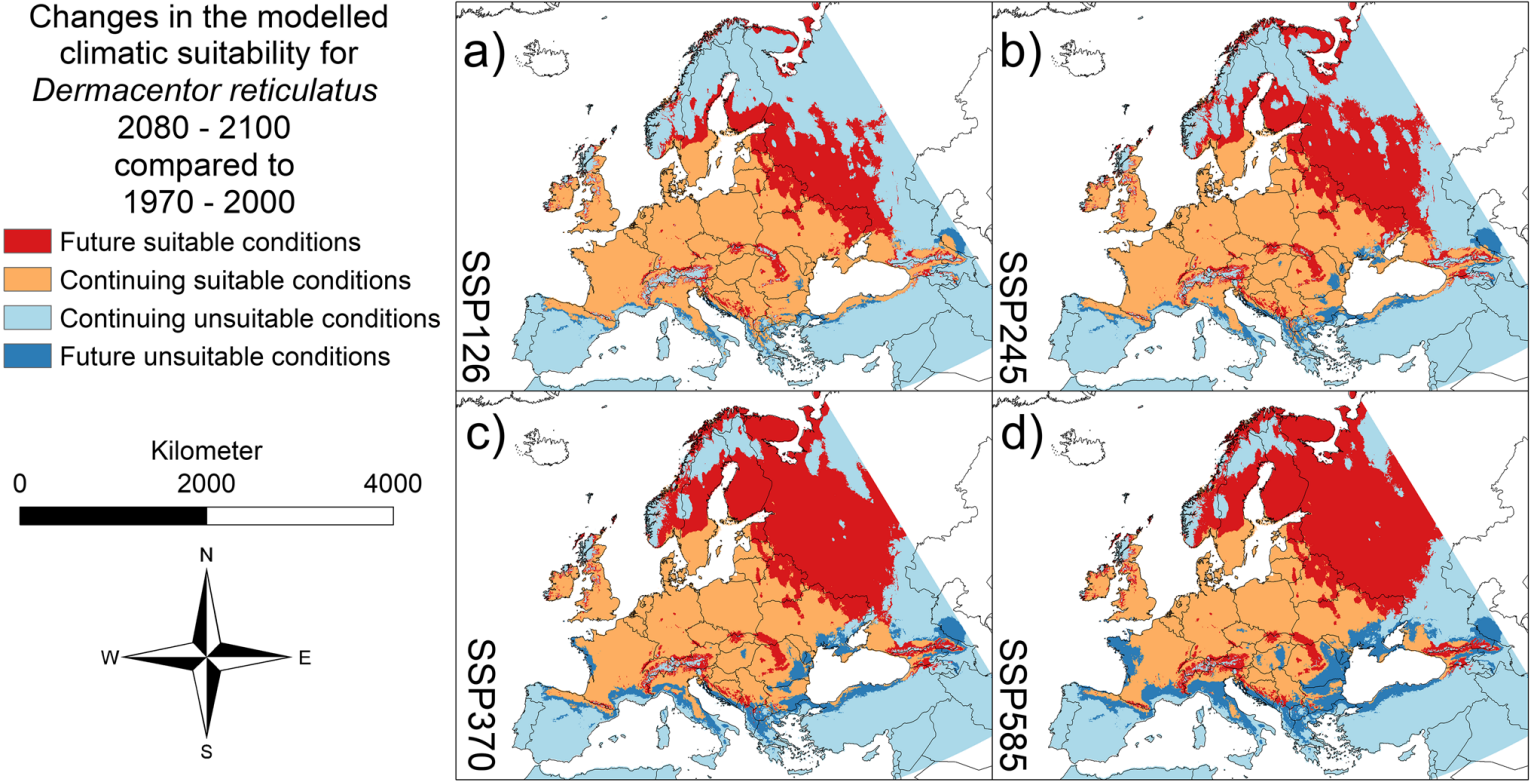
Projected future changes for *Dermacentor reticulatus* until 2080–2100. **a** SSP 126. **b** SSP 245. **c** SSP 370. **d** SSP 585. In dark blue: area projected as suitable under current climatic conditions but unsuitable under future climatic conditions (i.e., potential extinction). In light blue: area projected as unsuitable under current climatic conditions as well as under future climatic conditions (i.e., stable absence). In orange: area projected as suitable under current climatic conditions as well as under future climatic conditions (i.e., stable range). In red: area projected as unsuitable under current climatic conditions but suitable under future climatic conditions (i.e., potential new range). AUC = 0.8333 (average over 10 replicates using cross-validation, standard deviation = 0.001113603). Threshold to transform the logistic model output: 0.3816 (10% omission rate threshold). Maps were built using ESRI ArcGIS (Release 10.7, www.esri.com). Projection: Europe Albers Equal Area Conic

**Fig. 3:**
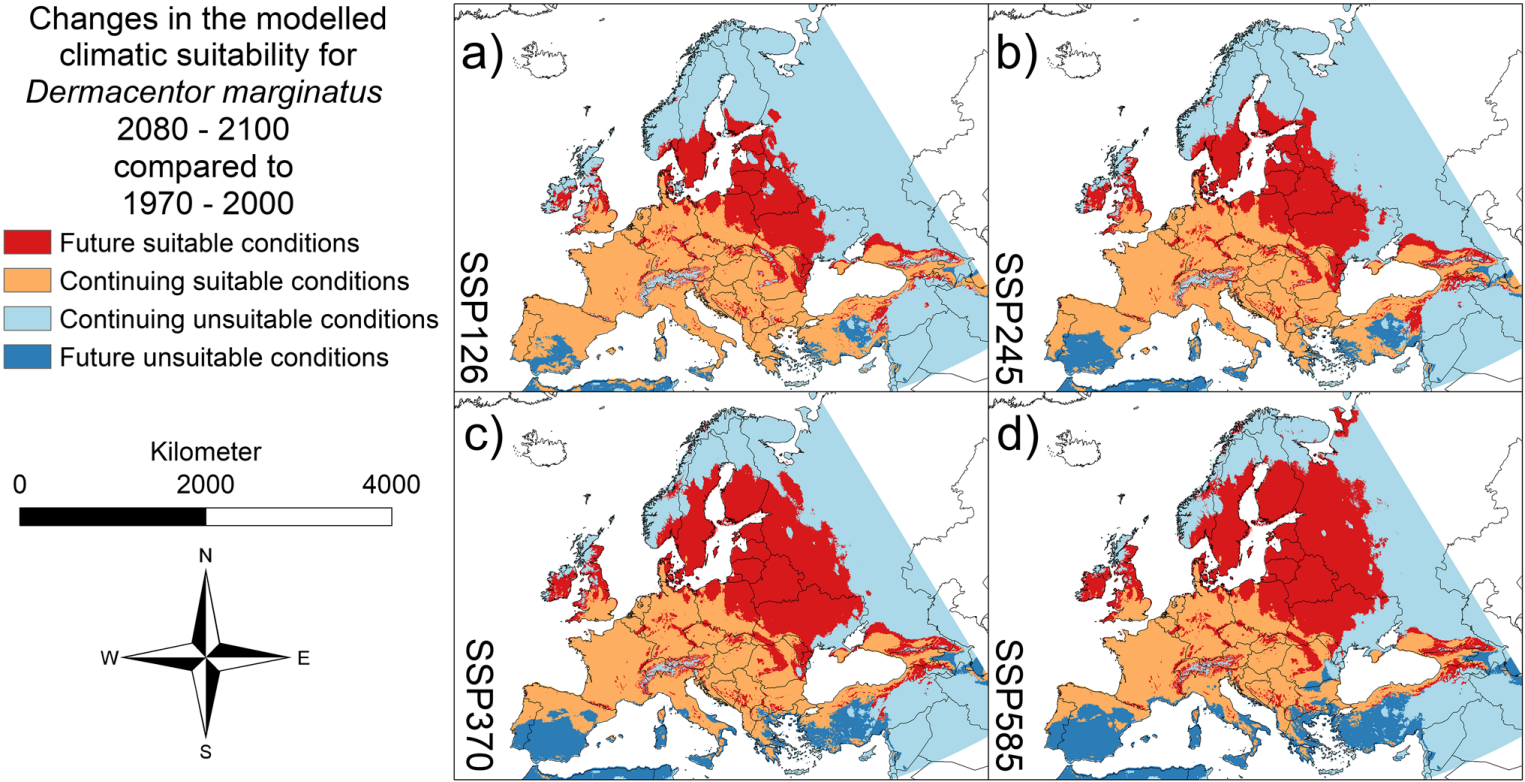
Projected future changes for *Dermacentor marginatus* until 2080–2100. **a** SSP 126. **b** SSP 245. **c** SSP 370. **d** SSP 585. In dark blue: area projected as suitable under current climatic conditions but unsuitable under future climatic conditions (i.e., potential extinction). In light blue: area projected as unsuitable under current climatic conditions as well as under future climatic conditions (i.e., stable absence). In orange: area projected as suitable under current climatic conditions as well as under future climatic conditions (i.e., stable range). In red: area projected as unsuitable under current climatic conditions but suitable under future climatic conditions (i.e., potential new range). AUC = 0.8229 (average over 10 replicates using cross-validation, standard deviation = 0.001121953). Threshold to transform the logistic model output: 0.4298 (10% omission rate threshold). Maps were built using ESRI ArcGIS (Release 10.7, www.esri.com). Projection: Europe Albers Equal Area Conic

**Fig. 4:**
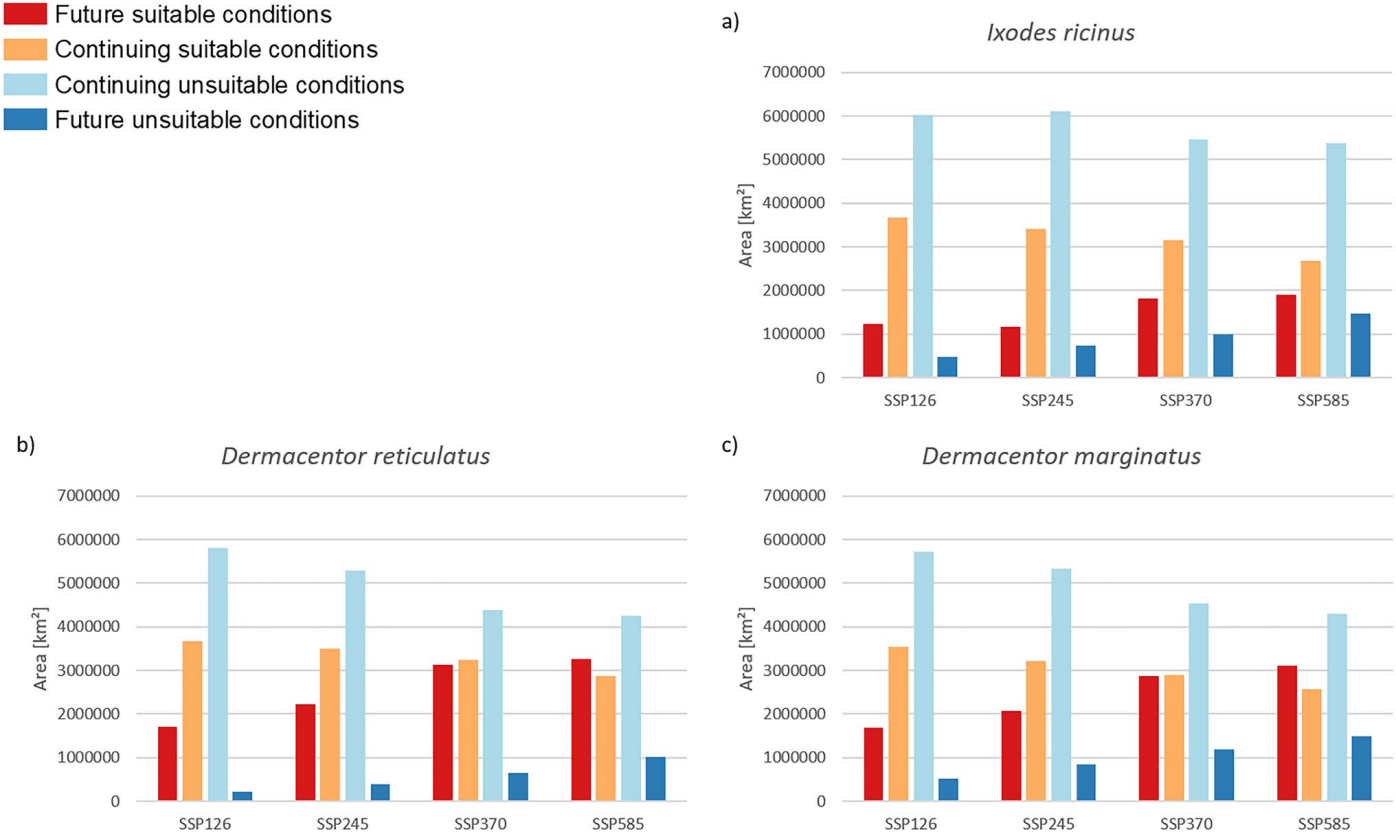
Area projected as suitable or unsutable under current and future (2081–2100) climatic conditions (km^2^) for the three tick species in comparison. **a** *Ixodes ricinus*. **b** *Dermacentor reticulatus*. **c** *D. marginatus*. The corresponding maps are shown in Figs. 1–3 in the main document. Future suitable conditions refers to the area (km^2^) projected as unsuitable under current climatic conditions but suitable under future climatic conditions (i.e., potential new range). Continuing suitable conditions refers to area (km^2^) projected as suitable under current climatic conditions as well as under future climatic conditions (i.e., stable presence). Continuing unsuitable conditions refers to area (km^2^) projected as unsuitable under current climatic conditions as well as under future climatic conditions (i.e. stable absence). Future unsuitable conditions refers to the area (km.^2^) projected as suitable under current climatic conditions but unsuitable under future climatic conditions (i.e., potential extinction)

**Fig. 5:**
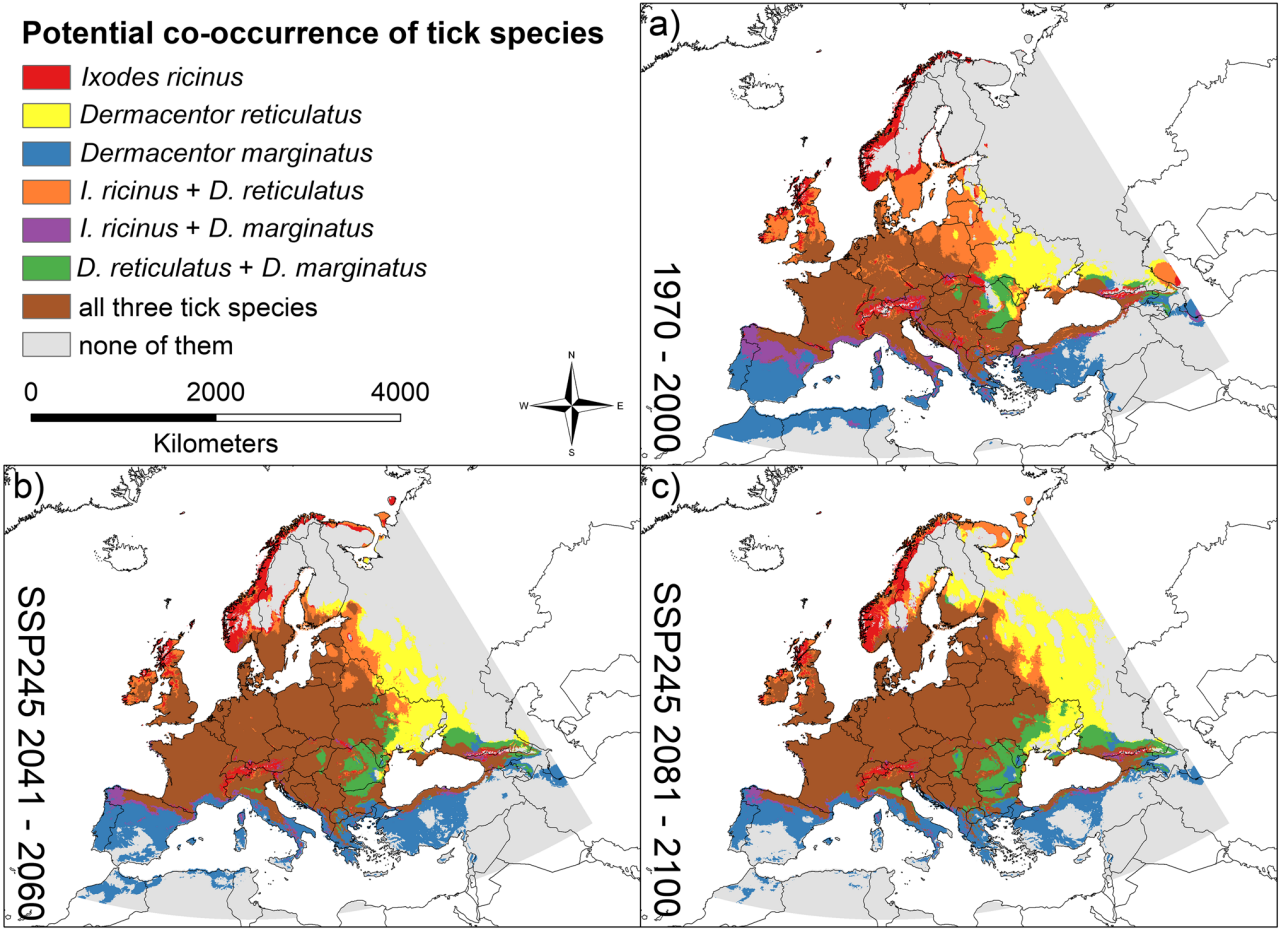
Potential co-occurrence under current and future climatic conditions. **a** Under near current climatic conditions (1970–2000). **b** Under projected future climatic conditions (exemplarily for SSP 245) for the period 2041–2060. **c** Under projected future climatic conditions (SSP 245) for the period 2081–2100. Colors indicate areas where climatic suitability is projected for the respective species; for non-mentioned species (“none of them”), the area is climatically unsuitable according to the modelling results. The thresholds to transform the logistic model output (10% omission rate threshold) are as follows: 0.3368 for *Ixodes ricinus*, 0.3816 for *Dermacentor reticulatus*, and 0.4298 for *D. marginatus*. Maps were built using ESRI ArcGIS (Release 10.7, www.esri.com). Projection: Europe Albers Equal Area Conic. (A hatch-based version of this figure is additionally provided in the Supplementary Material: Figure S11.)

The dichotomous Maxent results were further processed according to the research questions. Using the ESRI ArcMap raster calculator (ESRI 2018), we generated maps to identify areas where the modelled habitat suitability (suitable or not) differs between current and future climate conditions. Specifically, we identified potential new ranges and potential loss of habitat (Figs. 1, 2 and 3) and quantified the respective areas (Fig. 4a–c and Table S2 in the Supplementary Material) using the calculate geometry function with the Albers Equal Area Conic projection for Europe in ESRI ArcMap (ESRI 2018). In addition, we identified areas where climatic conditions are projected to be suitable for more than one species, i.e. a potential co-occurrence of the considered tick species might be possible (Fig. 5 and Figure S11 in the Supplementary Material). We also compared Maxent percent contribution values for the six predictor variables between the three tick species (Figure S12 in the Supplementary Material) to assess which main factors may drive the spatial distribution patterns of the species.

All maps were created using ArcGIS® software by Esri (ESRI 2018).

## Results

The modelled habitat suitability under near current climatic conditions reflect the distributional patterns of occurrence records well for all three tick species (Figures S3–S5 a) and Figure S1 in the Supplementary Material), indicated by high AUC values: AUC = 0.79, standard deviation (SD) = 6.4 × 10^−4^ for *I. ricinus*, AUC 0.83, SD = 1.1 × 10^−3^ for *D. reticulatus* and AUC = 0.82, SD = 1.1 × 10^−3^ for *D. marginatus*).

According to our modelling results, all three tick species will benefit from the projected climate changes in Europe (Figs. 1–3). For all three species, the modelling results project a range expansion towards the north-east, while habitat losses will occur in the Mediterranean region. The projected habitat gain exceeds the projected loss in each case, which means that larger areas are projected as climatically suitable for all three species in the future (Fig. 4a–c and Table S2 in the Supplementary Material). For all three species, the comparably largest range expansion is modelled under the SSP585 scenario, followed by the SSP370 scenario (Fig. 4a–c and Table S2 in the Supplementary Material). For the two species of the genus *Dermacentor*, the area with suitable climatic conditions in the future as well as the area that will no longer be suitable in the future is increasing, in ascending order of the scenarios, i.e., SSP126, SSP245, SSP370, and SSP 585 (Fig. 4b, c and Table S2 in the Supplementary Material).

Projected range shifts due to climate change will also cause changes in species co-occurrence patterns (Fig. 5). *Dermacentor marginatus* is modelled to find suitable climatic conditions in many parts of the Mediterranean and may occur furthest south. According to our results, all three tick species will find suitable climatic conditions in large parts of Central Europe in the future. The area climatically suitable for all three species is projected to expand north-eastwards in the future.

Among the considered six climatic variables, the minimum temperature of the coldest month (bio06) contributed most to the Maxent models (Figure S12 in the appendix) for all species. Temperature seasonality (bio04) and precipitation seasonality (bio15) are of relevance for *Ixodes ricinus,* and in addition, precipitation seasonality (bio15) is important for *D. reticulatus.*

## Discussion

The aim of this study was to examine the effects of projected climate change on the habitat suitability of three common tick species in Europe. The models were based on a comprehensive occurrence data set and account for uncertainties in the assumptions about potential future developments by applying the most recent scenarios on future climatic conditions. The results may be used as a component for risk assessments of tick-borne disease outbreaks.

The modelled habitat suitability under near current climatic conditions is largely in good accordance with the reported distributional patterns of *I. ricinus*, *D. reticulatus*, and *D. marginatus*. Small regional discrepancies can be found, for example, for *I. ricinus* in Portugal and North Africa, where the species has been reported several times, but according to the model finds only a low climatic suitability. In Spain, the morphologically similar species *Ixodes inopinatus* occurs (Estrada-Peña et al. 2014), and misidentified *I. inopinatus* might account for some records for *I. ricinus*.

According to the distribution maps provided by the European Centre for Disease Prevention and Control (ECDC) that are available for *I. ricinus* (ECDC 2020b) and *D. reticulatus* (ECDC 2020a), the observed distribution of the species goes even beyond the area modelled as climatically suitable in our results. This can be partly attributed to the different scales of the data included. While our models use point distribution data (and thus also account for abundances), the ECDC data refer to species’ presence or absence in the administrative areas. The main distribution area of the three species is well reflected in our models (possibly slightly conservatively); so our approach is appropriate to estimate future trends and possible climate change-induced range shifts.

Our modelling results clearly show that all three species will all benefit from the projected climatic changes, with a clear range expansion under future climatic conditions in Europe. According to the results of the MESS analysis (Figure S9 in the Supplementary Material), there are only small environmental differences between model training and model projection (extrapolation) data (restricted to areas in northern Africa). The projected range expansions for the tick species is consistent with the observed range changes and local increases in abundance in recent decades (Dautel et al. 2006; Gray et al. 2009; Hvidsten et al. 2020; Medlock et al. 2013; Rubel et al. 2016). Our results are also largely consistent with the results of other modelling studies (e.g., Porretta et al. 2013 for *I. ricinus*, Williams et al. 2015 for *I. ricinus* and *D. marginatus*). Smaller local differences may be due to different underlying distributional data, different sets of predictor variables or different scenarios for future climatic conditions. Similar to Porretta et al. (2013), the extreme scenarios (i.e., A2, 585) yield comparatively lower modelled habitat suitability values, but with range expansion when interpreting the binary results. This could be taken as a hint that very high temperatures could have a limiting effect on the distribution of *I. ricinus*.

Conditions of temperature play a crucial role in terms of distribution patterns of ticks as they determine survival, development, and questing behavior (Lindgren et al. 2000; Tomkins et al. 2014). Among the considered bioclimatic variables, the minimum temperature of the coldest month (bio06) contributed most to the model for all three species. This is in accordance with the results of e.g. Porretta et al. (2013) who for *I. ricinus* also identified bio06 as the most contributing variable and a plausible result in view of ticks being ectothermal arthropods sensitive to low temperature (Gray et al. 2009). Low temperature in winter can hinder range expansion towards northern and eastern Europe and has been identified as an important limiting factor towards Northern and Eastern Europe for mosquitoes as well (Cunze et al. 2016b). *Ixodes ricinus* is described as comparably cold-resistant and can be found further north than the other two species today. Acclimatized individuals have been found to survive a 24-h exposure at temperatures from − 14.4 to − 18.9 °C, whereas a longer exposure is considered harmful (Dautel and Knülle 1997). However, it is assumed that variables as temperature sums (defined as accumulated measures over a certain period of time exceeding a threshold value) may be more determining for the survival and development of *I. ricinus* than extreme temperatures (Gray et al. 2009). *Dermacentor reticulatus* is also considered to be cold-resistant (Földvári et al. 2016). In contrast, *D. marginatus* is assumed to be cold-sensitive, showing a high mortality at − 15 °C within a short period of time in the laboratory (Dörr and Gothe 2001). This is consistent with its distribution and occurrence records ranging furthest south among the three tick species (Drehmann et al. 2020). Under field conditions, ticks generally search for protected places to spend their diapause over the winter, which allows them to survive even in areas where temperatures fall below the survival thresholds identified under laboratory conditions.

High temperatures in summer may also be a potential limiting factors for tick distribution, but especially in combination with humidity. In addition to temperature, humidity is of major importance for tick species during their off-host phases. *Ixodes ricinus* requires a relative humidity of at least 80% (Medlock et al. 2013). In our models, we consider precipitation variables (due to better data availability) as proxies for humidity. Annual precipitation and the precipitation of the driest month only contributed little to the modelling results whereas the variable contribution of precipitation seasonality (bio15) was about 21% for *I. ricinus* and *D. reticulatus*.

It is assumed that the increase in vector-borne diseases can be explained to a large part by climate change. However, the underlying relationships are complex and do not follow a simple cause and effect concept (Whitehorn and Yacoub 2019). Moreover, other factors also play a role for tick species to occur in a certain area and are discussed in the following section.

By their nature, models are subject to a number of uncertainties. However, this does not preclude niche modelling from being a very useful tool with regard to, e.g., assessing the risk of tick-borne disease transmission, and using the results to make monitoring, surveillance and, if necessary, control more efficient. Evenso one needs to be aware of some limitations when interpreting the results.

A particularly sensitive issue is the quality of distributional data, which is most often affected by sampling bias. Overall, the effect of sampling bias is difficult to assess due to the lack of reference data sets of an unbiased species’ distribution against which an independent evaluation could be made. Ribeiro et al. (2019) examined the effect of data quality on modelling results exemplified by *I. ricinus* in Scotland. They conclude that considering several data sources could enhance coverage and point out the importance of being aware of the uncertainties and limitations of modelling associated with lower data quality.

We attempted to address a potential sampling bias in the data by using distribution data from more than one source, plotting the available data on a map and visually checking for plausibility, as well as spatially thinning the data.

Another crucial aspect of reliable modelling is the consideration of all relevant factors that shape the distribution patterns of the considered species. We here focused only on climatic conditions as climatic conditions are considered the most important factor for the distributional patterns on a continental scale (Peterson 2011). Another advantage is that there are scenarios about future conditions for the climatic factors and thus projections about the future distribution of the species can be obtained.

Land cover and vegetation cover are also important factors that shape the distribution patterns of tick species because they are indirectly affected by climatic conditions, including also edaphic conditions, and in particular, they are anthropogenically determined. For example, it has been reported that *D. reticulatus* benefits from the rise in fallow lands as a result of EU agricultural policies (Gray et al. 2009). *Dermacentor reticulatus* generally shows a high association to certain habitat types as, e.g., alluvial forests, swamps, lake shores and riverbanks but also drier habitats as woodland edge, heathland, grassland, sand dunes and suburban forests and parks (Medlock et al. 2017). However, in our models, we did not account for land cover based on the assumption that at a continental level, climatic conditions are the most important drivers shaping spatial distribution patterns (Peterson 2011). Moreover, ticks cover a wide range of different habitat types and favorable small-scale structures (i.e., litter layer) should be available in many habitats in most climatic zones.

Biotic interactions also play an important role and have recently been incorporated into a model for *I. ricinus* by including dispersal data from vertebrate hosts (Fernández-Ruiz and Estrada-Peña 2020). These interactions can also change if host species migrate into new areas and therefore influence spatial distribution patterns of tick species. However, such interactions can only be included indirectly (via occurrence data) in correlative distribution models.

Another biotic factor that is not included directly in our models is availability of hosts. All three species have a broad spectrum of hosts (Buczek et al. 2015; Estrada-Peña and La Fuente 2017; Földvári et al. 2016). Preferred host species differ between the life stages (Stanek et al. 2012). The larvae feed on small mammals (especially rodents), birds, and reptiles. Nymphs feed on medium-sized mammals such as foxes but also on birds and humans. In the adult stage, larger mammals such as deer, sheep, dogs, and humans serve as hosts (Herrmann and Gern 2015). However, since a large number of hosts are available, host availability is unlikely to be a limiting factor for the spatial distribution of ticks in Europe (Estrada-Peña and La Fuente 2017) and the recent range expansion of *D. reticulatus* could be related to an increasing number of available hosts, such as deer (Gray et al. 2009; Mierzejewska et al. 2015).

In the same way, the question of whether species can migrate fast enough to keep pace with the climate change induced shifts in habitat suitability is not accounted for in our approach and must be kept in mind when discussing potential future distribution patterns (Cunze et al. 2013). Mild winters may elongate the duration of the activity periods of ticks (Whitehorn and Yacoub 2019), and rising temperatures could shorten the duration of tick development cycles and enhance local abundances. Thus, apart from the factors described above, further studies on physiological limits and life-cycle developments under changing temperatures are necessary to assess the future risk of human infection with tick-borne diseases and to be able to perform reliable risk assessments for tick-disease occurrences.

## Conclusion

According to our results, the habitat suitability for *Ixodes ricinus*, *Dermacentor reticulatus*, and *Dermacentor marginatus* tends to increase in Europe under future climatic conditions, with projected area expansions towards Eastern Europe. All three tick species are modelled to find suitable climatic conditions in Central Europe in the future and co-occurring changes in the diversity and local abundances may increase the risk of human infections with diseases transmitted by these species. A close surveillance of the three species and a monitoring following standardized guidelines is warranted.

## Supplementary information

Below is the link to the electronic supplementary material.

Supplementary file1 (PDF 12667 KB)
