# Supplementary material for: Ticks on the move—climate change-induced range shifts of three tick species in Europe: current and future habitat suitability for Ixodes ricinus in comparison with Dermacentor reticulatus and Dermacentor marginatus
Source: Parasitol Res. 2022 Jun 1;121(8):2241–52. doi: 10.1007/s00436-022-07556-x (PMC9279273; doi:10.1007/s00436-022-07556-x)
Supplement: Supplementary file 1 — Supplementary file1 (PDF 12667 KB) [file 436_2022_7556_MOESM1_ESM.pdf]

Ticks on the move – climate change-induced range shifts of three tick species in Europe

Sarah Cunze, Gustav Glock, Judith Kochmann, Sven Klimpel

Occurrence data

Table S1: Numbers of occurrence records (raw data) by species and source. Estrada-Peña et al. 2016 (https://doi.org/10.5061/dryad.2h3f2); GBIF 2020a (https://doi.org/10.15468/dl.8tr4te), GBIF 2020b (https://doi.org/10.15468/dl.qyk2m3), GBIF 2020c (https://doi.org/10.15468/dl.tt6rkm) and Rubel et al. 2016 (https://doi.org/10.1016/j.ttbdis.2015.10.015).

Ixodes ricinus	*Dermacentor reticulatus*	*Dermacentor marginatus*	Reference
1196	29	378	Estrada-Peña et al. 2016
2550			GBIF 2020a
		104	GBIF 2020b
	201		GBIF 2020c
	719		Rubel et al. 2016
		826	Rubel et al. 2016
**3746**	**949**	**1308**	

Ticks on the move – climate change-induced range shifts of three tick species in Europe

Sarah Cunze, Gustav Glock, Judith Kochmann, Sven Klimpel

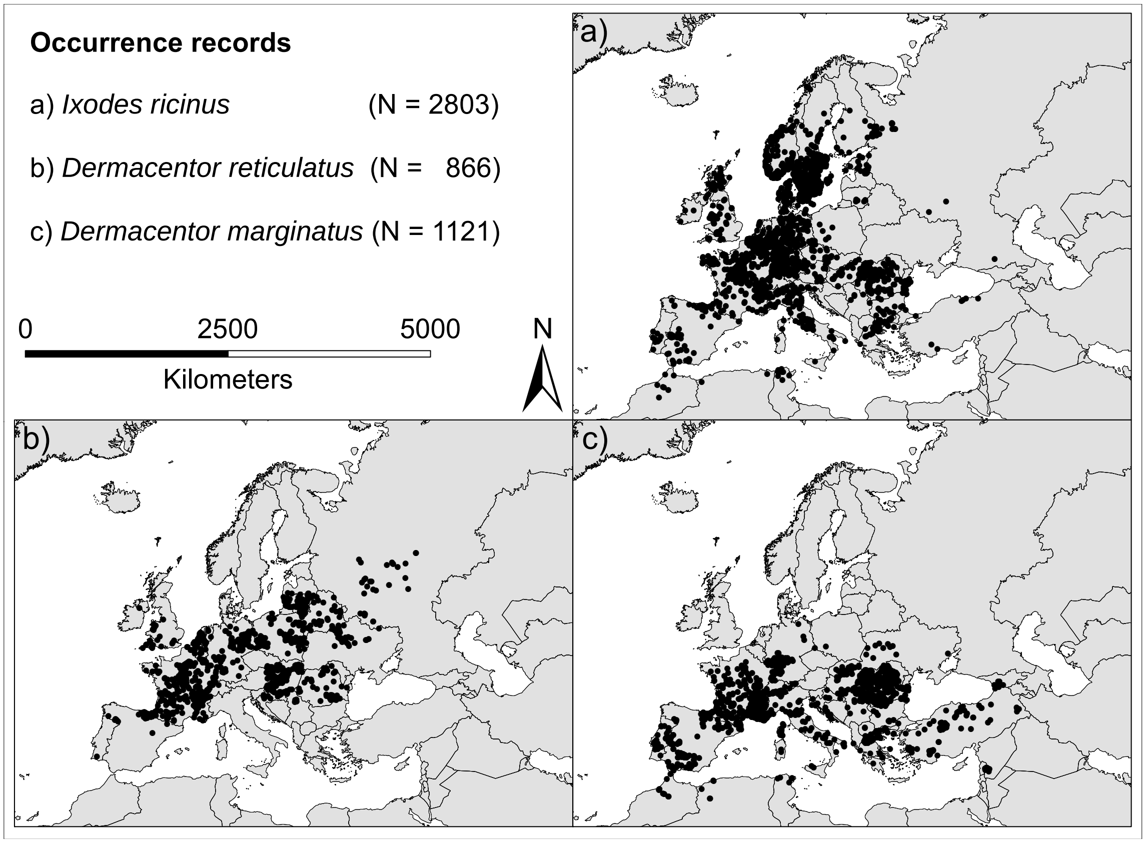

Figure S1: Occurrence data used for modelling. a) *Ixodes ricinus*, b) *Dermacentor reticulatus*, c) *Dermacentor marginatus*. Maps were built using ESRI ArcGIS (Release 10.8, www.esri.com). Projection: Europe Albers Equal Area Conic.

Ticks on the move – climate change-induced range shifts of three tick species in Europe

Sarah Cunze, Gustav Glock, Judith Kochmann, Sven Klimpel

Environmental variables

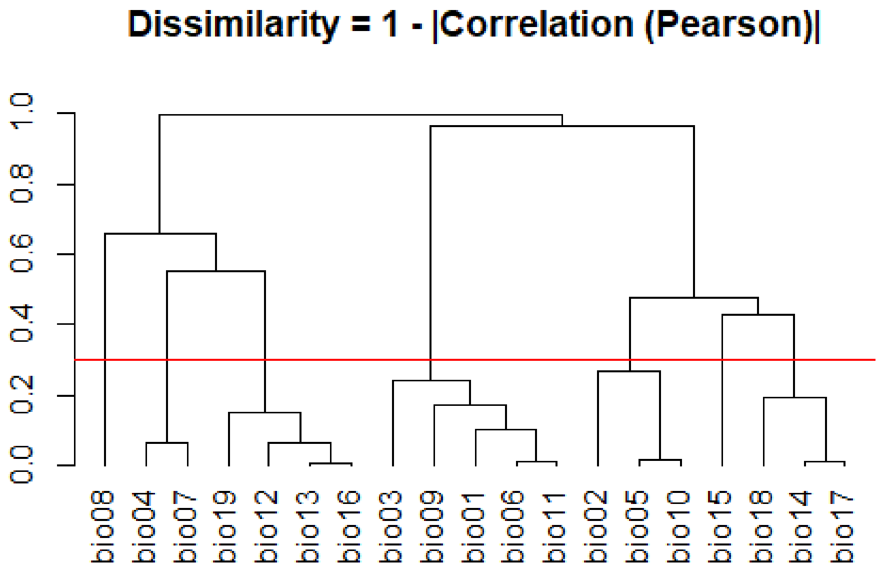

Figure S2: Variable selection. For the 19 bioclimatic variables provided by worldclim, we generated a dendrogram based on the “1 - Pearson correlation coefficients” – dissimilarity (method: complete linkage). We built groups of interrelated variables (with threshold of 0.3 – red line). After excluding bio08, bio09, bio18 and bio 19, which are effected by artefacts, we chose one representative of each group that is considered ecologically relevant and easy to interpret. Specifically we chose: bio04 (temperature seasonality), bio05 (maximum temperature of warmest month), bio06 (minimum temperature of the coldest month), bio12 (annual precipitation), bio14 (precipitation of the driest month) and bio15 (precipitation seasonality).

Ticks on the move – climate change-induced range shifts of three tick species in Europe

Sarah Cunze, Gustav Glock, Judith Kochmann, Sven Klimpel

Additional results

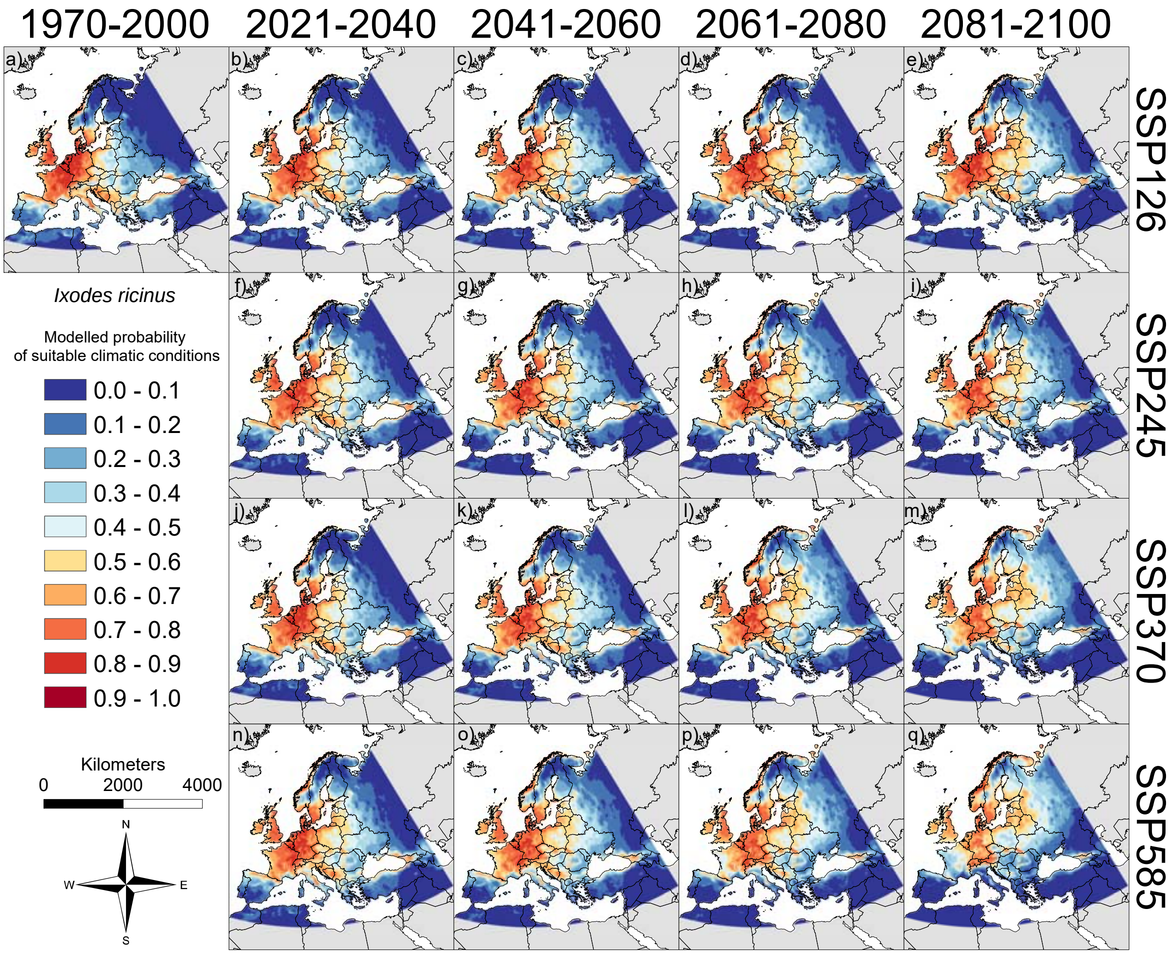

Figure S3: Modelled probability that climatic conditions are suited for *Ixodes ricinus* in Europe under current and future climatic conditions accounting for different SSP scenarios and time periods. Warmer colours indicate higher projected probability (AUC = 0.7917 +/- 0.000641959, unregularised test gain = 0.5913 +/- 0.03635144 – 10 replicates). Maps were built using ESRI ArcGIS (Release 10.8, www.esri.com). Projection: Europe Albers Equal Area Conic.

Ticks on the move – climate change-induced range shifts of three tick species in Europe

Sarah Cunze, Gustav Glock, Judith Kochmann, Sven Klimpel

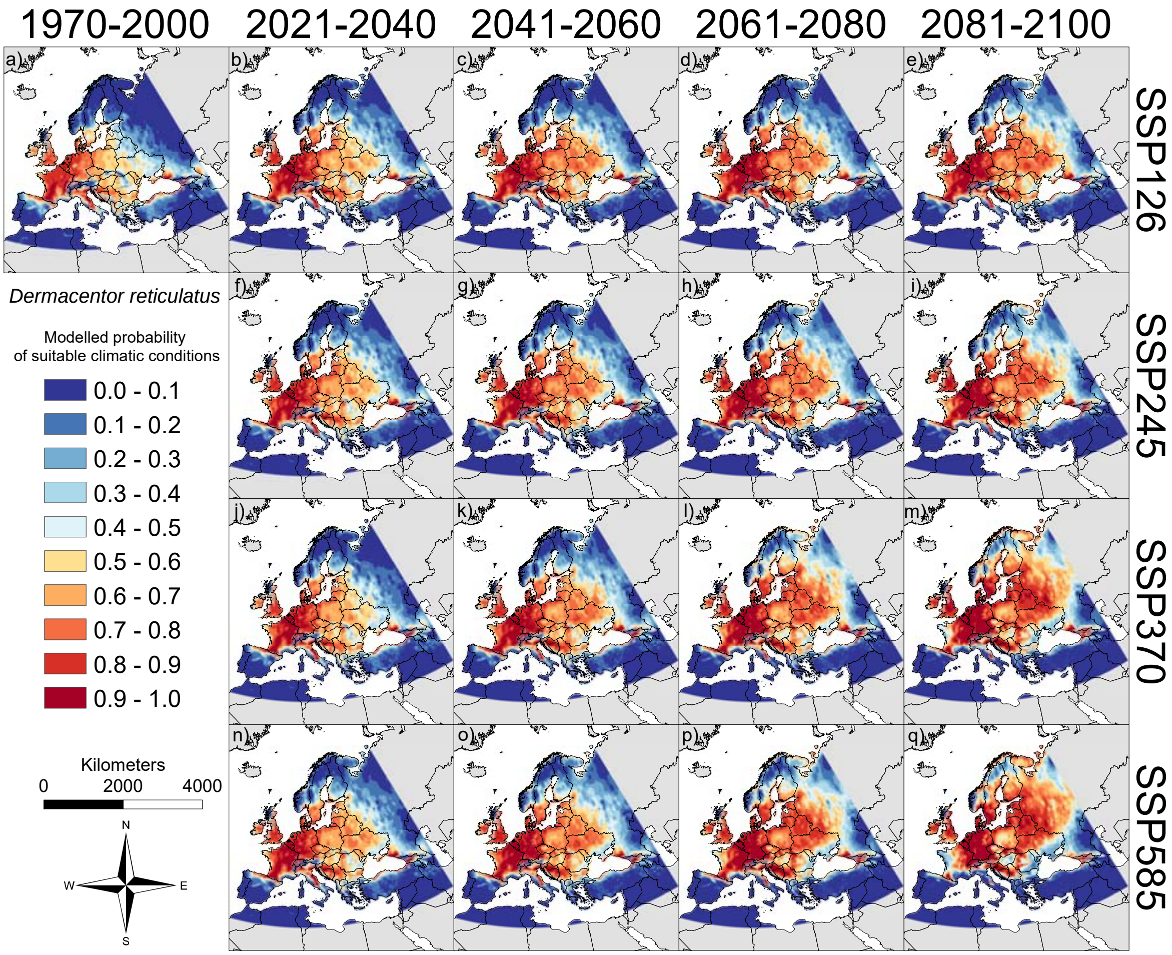

Figure S4: Modelled probability that climatic conditions are suited for *Dermacentor reticulatus* in Europe under current and future climatic conditions accounting for different SSP scenarios and time periods. Warmer colours indicate higher projected probability (AUC = 0.8333 +/- 0.001113603, unregularised test gain = 0.7807 +/- 0.00942708 – 10 replicates). Maps were built using ESRI ArcGIS (Release 10.8, www.esri.com). Projection: Europe Albers Equal Area Conic.

Ticks on the move – climate change-induced range shifts of three tick species in Europe

Sarah Cunze, Gustav Glock, Judith Kochmann, Sven Klimpel

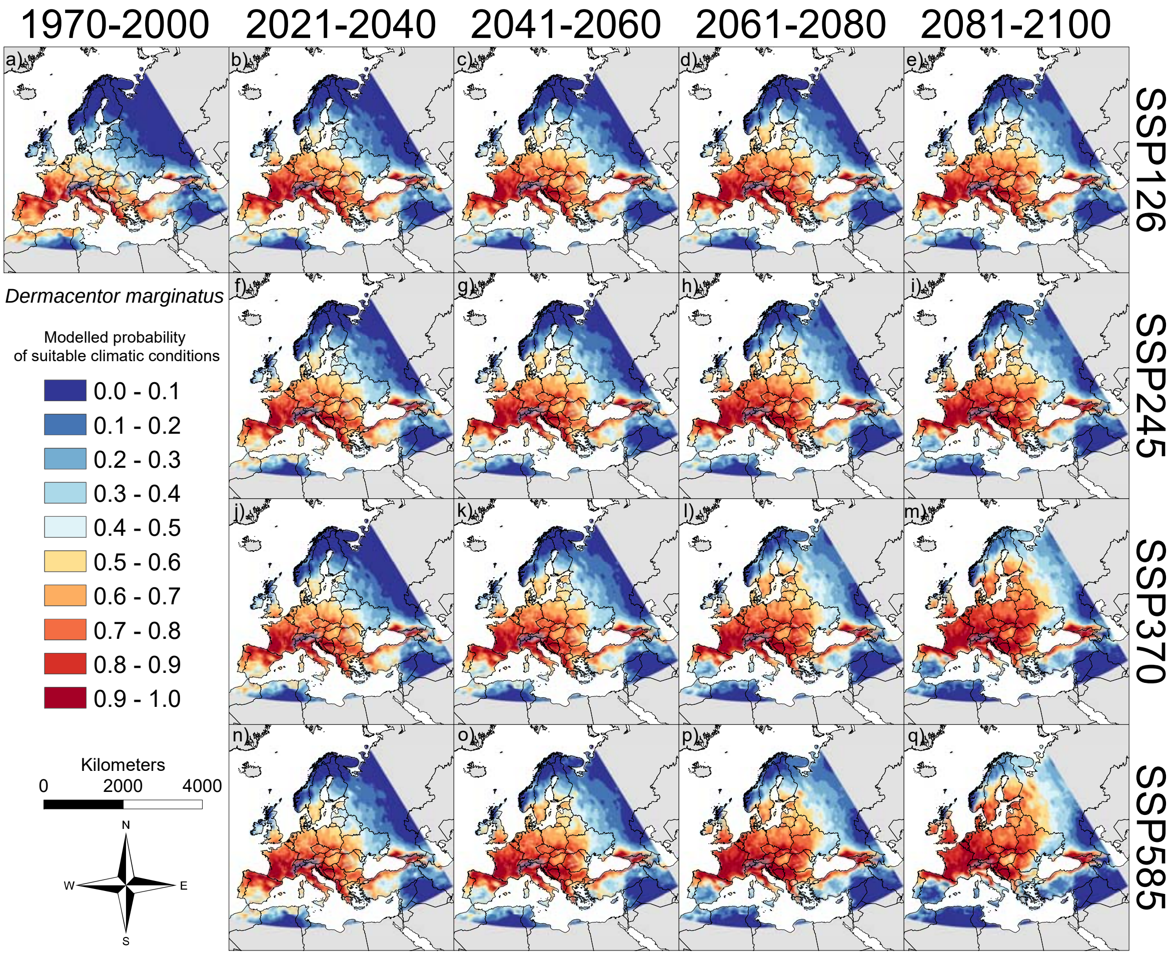

Figure S5: Modelled probability that climatic conditions are suited for *Dermacentor marginatus* in Europe under current and future climatic conditions accounting for different SSP scenarios and time periods. Warmer colours indicate higher projected probability (AUC = 0.8229 +/- 0. 001121953, unregularised test gain = 0. 7216 +/- 0.05255974 – 10 replicates). Maps were built using ESRI ArcGIS (Release 10.8, www.esri.com). Projection: Europe Albers Equal Area Conic.

Ticks on the move – climate change-induced range shifts of three tick species in Europe

Sarah Cunze, Gustav Glock, Judith Kochmann, Sven Klimpel

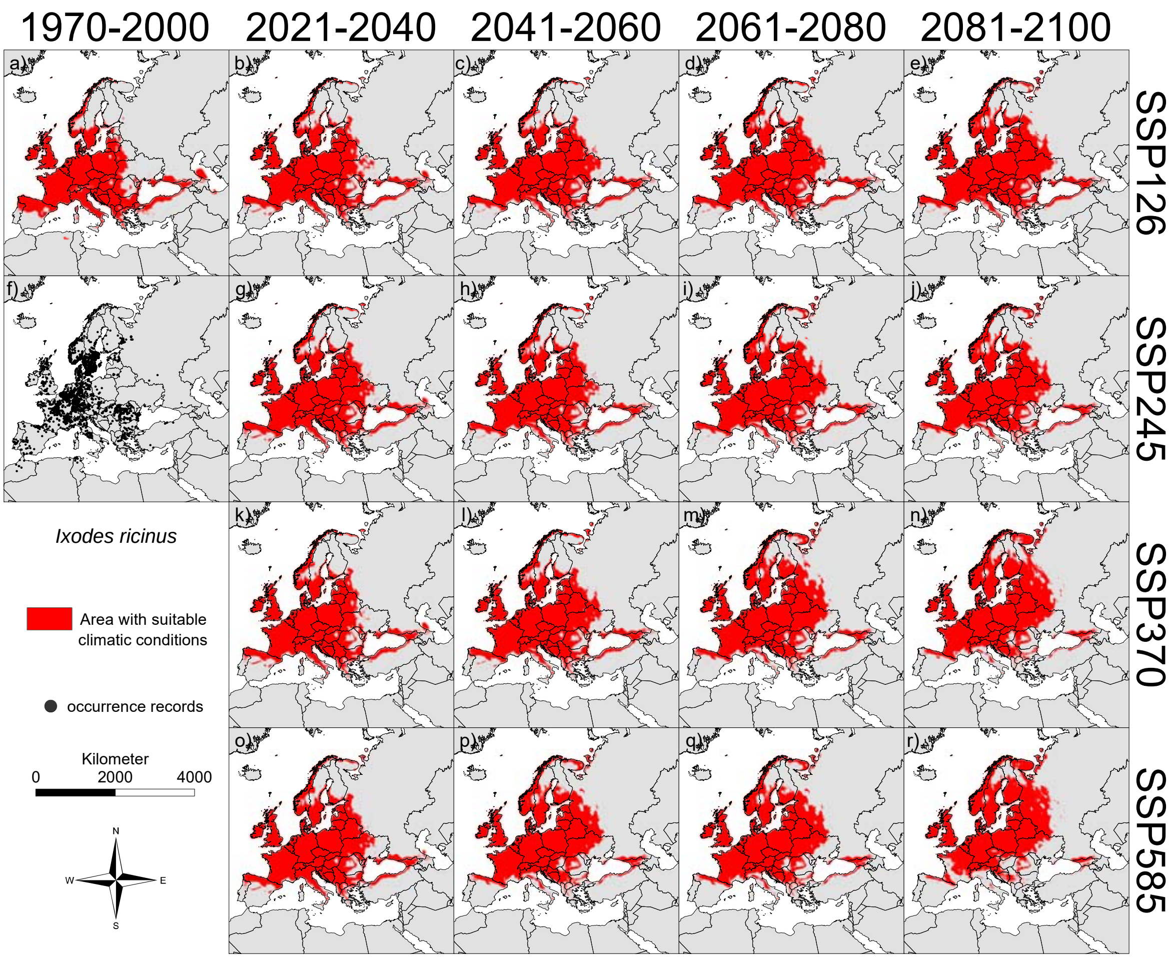

Figure S6: Area with modelled climatic suitability for *Ixodes ricinus* in Europe under current and future climatic conditions. Dichotomous modelling results using the 10% omission rate threshold (T = 0.3368). Maps were built using ESRI ArcGIS (Release 10.8, www.esri.com). Projection: Europe Albers Equal Area Conic.

Ticks on the move – climate change-induced range shifts of three tick species in Europe

Sarah Cunze, Gustav Glock, Judith Kochmann, Sven Klimpel

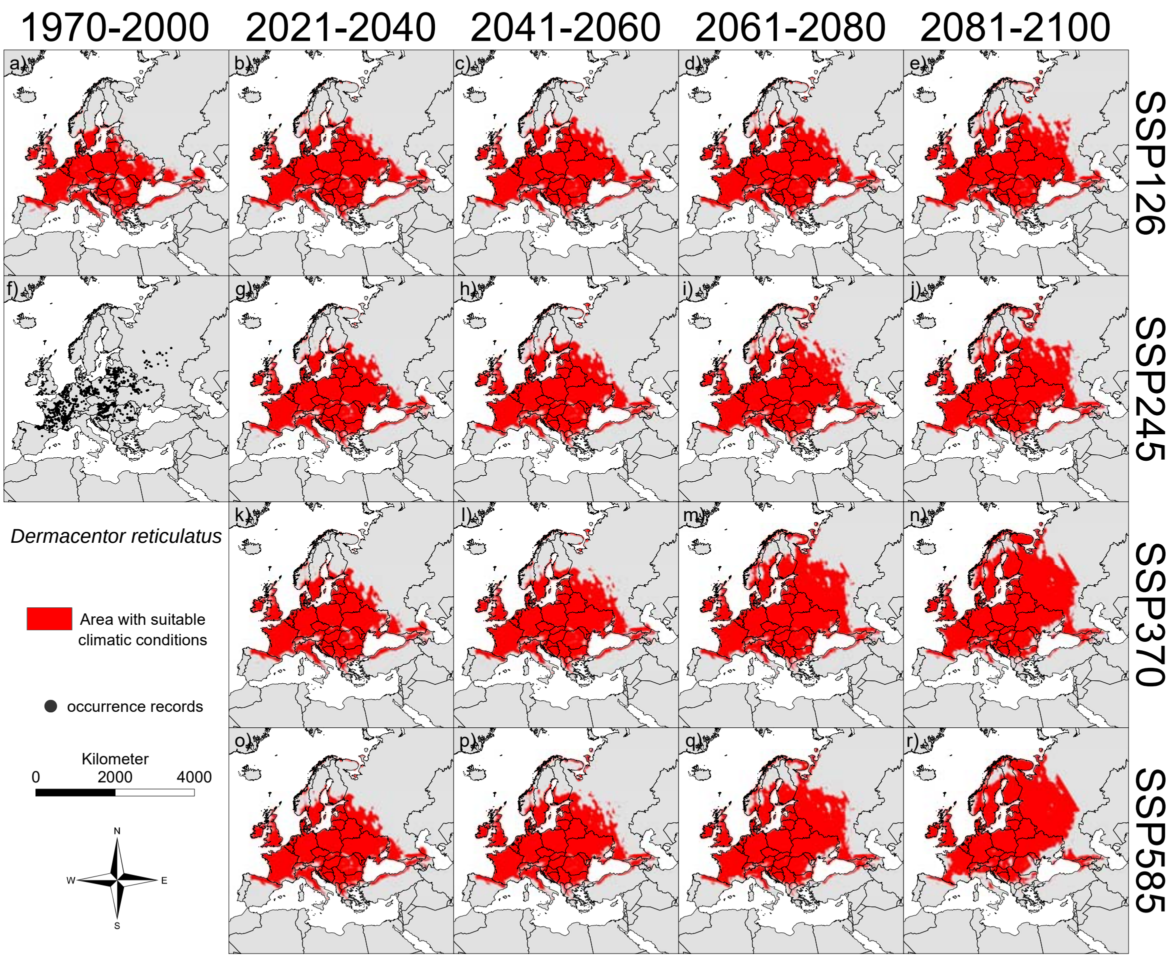

Figure S7: Area with modelled climatic suitability for *Dermacentor reticulatus* in Europe under current and future climatic conditions. Dichotomous modelling results using the 10% omission rate threshold (T = 0.3816). Maps were built using ESRI ArcGIS (Release 10.8, www.esri.com). Projection: Europe Albers Equal Area Conic.

Ticks on the move – climate change-induced range shifts of three tick species in Europe

Sarah Cunze, Gustav Glock, Judith Kochmann, Sven Klimpel

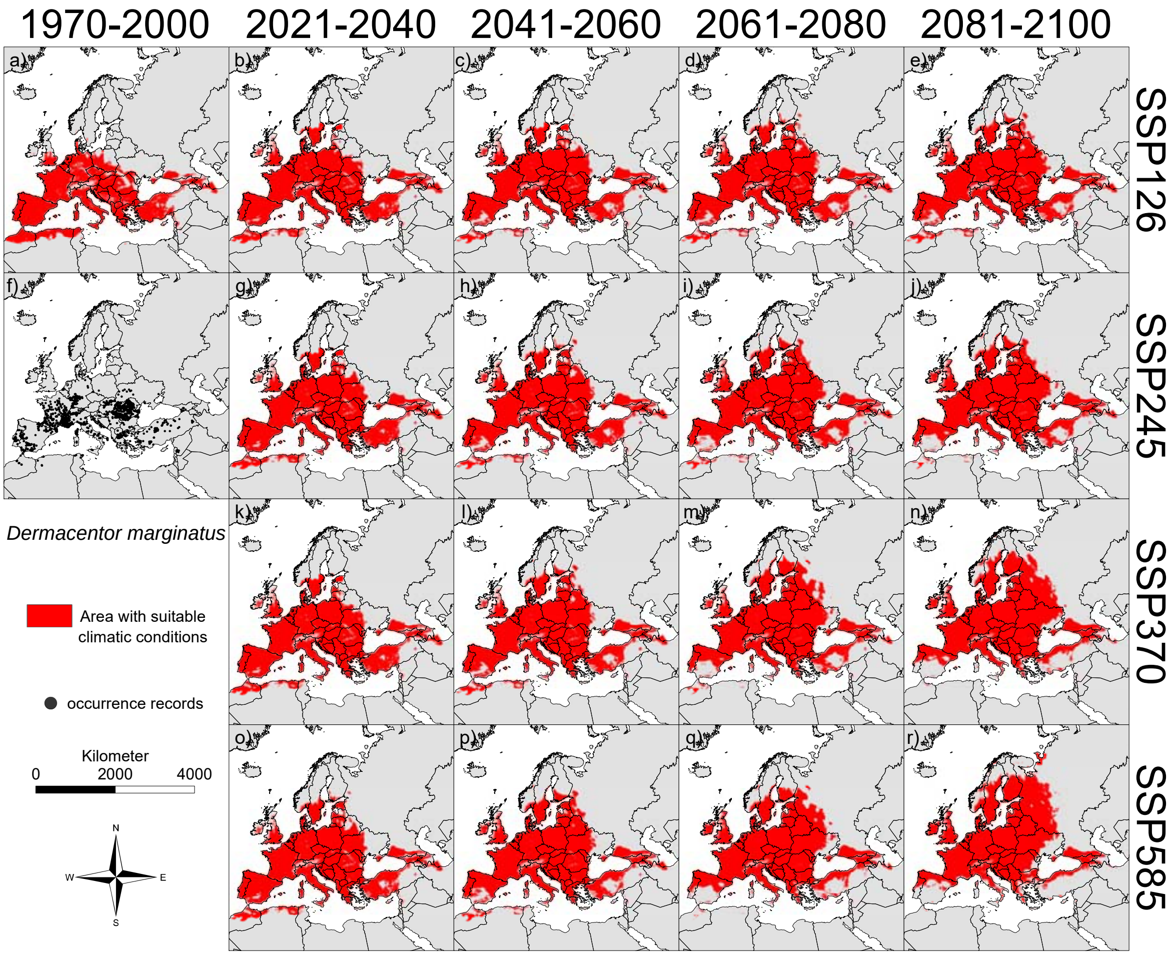

Figure S8: Area with modelled climatic suitability for *Dermacentor marginatus* in Europe under current and future climatic conditions. Dichotomous modelling results using the 10% omission rate threshold (T = 0. 4298). Maps were built using ESRI ArcGIS (Release 10.8, www.esri.com). Projection: Europe Albers Equal Area Conic.

Ticks on the move – climate change-induced range shifts of three tick species in Europe

Sarah Cunze, Gustav Glock, Judith Kochmann, Sven Klimpel

Table S2: Quantification of projected potential range changes [km²] for a) *Ixodes ricinus*, b) *Dermacentor reticulatus* and c) *D. marginatus* in Europe (cf. Figure 1.3). "Future suitable conditions" refers to the area [km²] projected as unsuitable under current climatic conditions but suitable under future climatic conditions (i.e. potential new range). "Continuing suitable conditions" refers to area [km²] projected as suitable under current climatic conditions as well as under future climatic conditions (i.e. stable presence). "Continuing unsuitable conditions" refers to area [km²] projected as unsuitable under current climatic conditions as well as under future climatic conditions (i.e. stable absence). "Future unsuitable conditions" refers to the area [km²] projected as suitable under current climatic conditions but unsuitable under future climatic conditions (i.e. potential extinction). The AUC-value refers to the average over 10 replicates using cross-validation +/- standard deviation. The threshold to transform the logistic model output was the 10% omission rate threshold. See Figure S9 in the Supplementary Material for a bar plot of the results.

**Area [km²]**	**SSP126**	**SSP245**	**SSP370**	**SSP585**
a) Ixodes ricinus	AUC = 0.7917 +/- 0.000641959, Threshold = 0.3368			
Future suitable conditions	1,235,391	1,159,487	1,807,509	1,892,781
Continuing suitable conditions	3,668,562	3,417,046	3,147,341	2,684,987
Continuing unsuitable conditions	6,026,242	6,103,370	5,455,814	5,370,348
Future unsuitable conditions	483,078	734,232	1,003,375	1,466,367
b) Dermacentor reticulatus	AUC = 0.8229 +/- 0.001121953, Threshold = 0.4298			
Future suitable conditions	1,700,162	2,221,718	3,134,222	3,267,978
Continuing suitable conditions	3,674,916	3,509,930	3,245,913	2,883,994
Continuing unsuitable conditions	5,815,644	5,295,235	4,383,977	4,250,759
Future unsuitable conditions	222,659	386,589	649,758	1,011,349
c) Dermacentor marginatus	AUC = 0.8333 +/- 0.001113603, Threshold = 0.3816			
Future suitable conditions	1,690,325	2,070,758	2,879,620	3,115,149
Continuing suitable conditions	3,542,654	3,214,374	2,885,468	2,578,350
Continuing unsuitable conditions	5,722,006	5,342,624	4,534,260	4,299,055
Future unsuitable conditions	528,393	855,595	1,183,930	1,490,465

Ticks on the move – climate change-induced range shifts of three tick species in Europe

Sarah Cunze, Gustav Glock, Judith Kochmann, Sven Klimpel

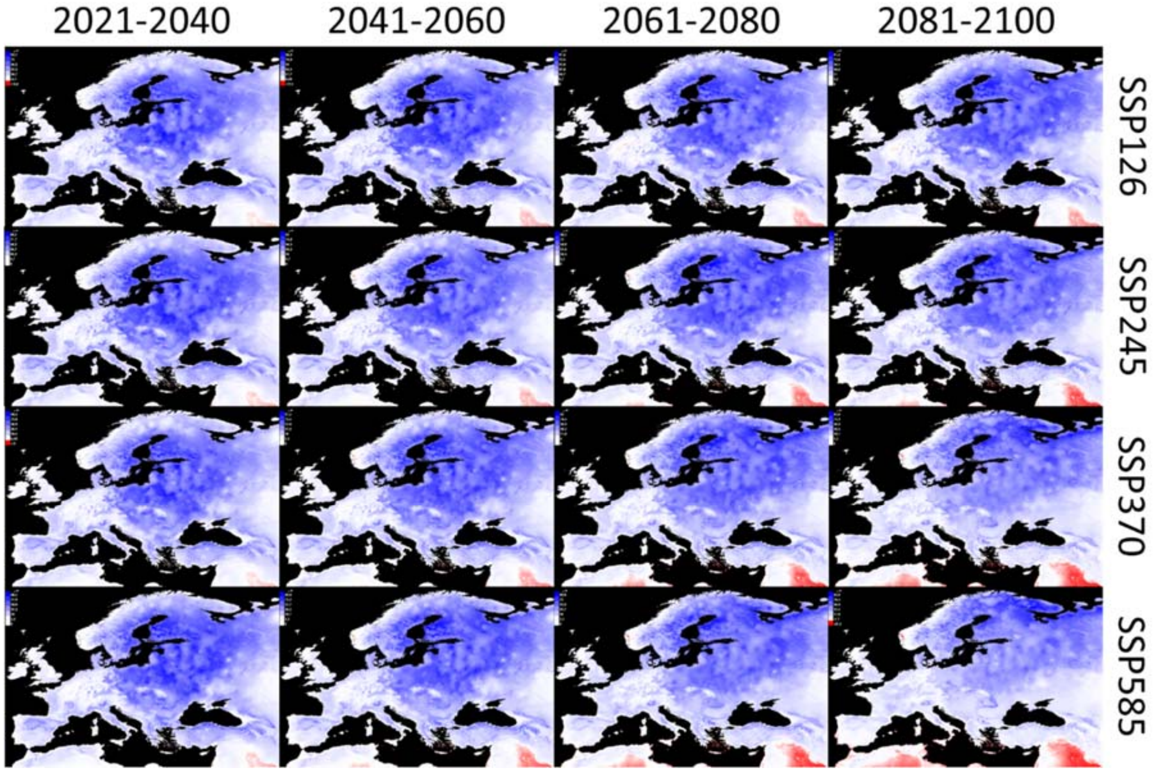

Figure S9: Results of the Maxent MESS analysis. Multidimensional environmental similarity surface (MESS) analysis indicates areas where novel climate conditions exist in the projection layers. Areas in red have one or more bioclimatic variables outside the range covered by the near current climatic conditions (1970 – 2000) used for model training. Modelling results in these areas should be treated with strong caution. Projection: WGS 1984.

Ticks on the move – climate change-induced range shifts of three tick species in Europe

Sarah Cunze, Gustav Glock, Judith Kochmann, Sven Klimpel

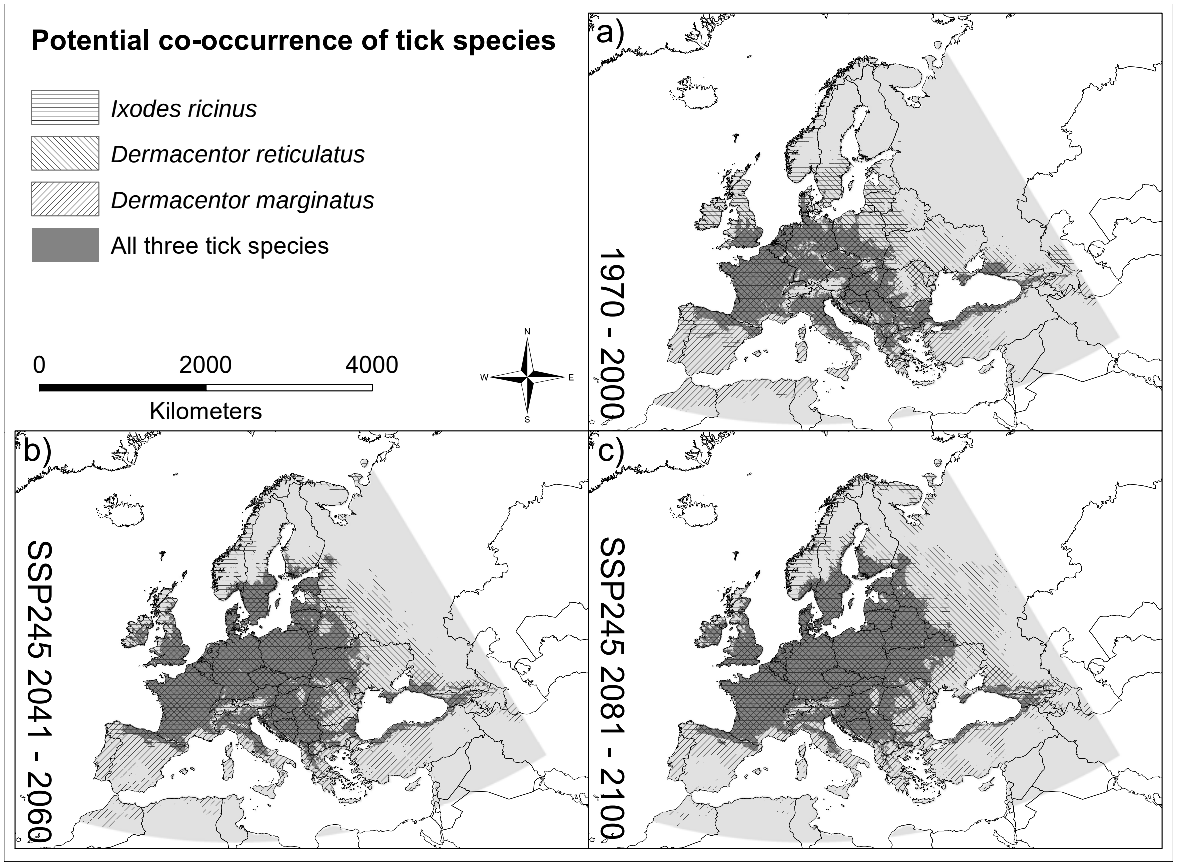

Figure S11: Potential co-occurrence under current and future climatic conditions. (Hatch-based Version of Figure 4 of the main document) a) under near current climatic conditions (1970-2000), b) under projected future climatic conditions (examplarily for SSP 245) for the period 2041-2060, c) under projected future climatic conditions (SSP 245) for the period 2081-2100. Hatchings indicate areas where climatic suitability for the respective species is projected, for non mentioned species the area is climatically unsuitable according to the modelling results. The thresholds to transform the logistic model output (10% omission rate threshold) are: 0.3368 for *Ixodes ricinus*, 0.3816 for Dermacentor reticulatus and 0.4298 for *D. marginatus*. Maps were built using ESRI ArcGIS (Release 10.8, www.esri.com). Projection: Europe Albers Equal Area Conic.

Ticks on the move – climate change-induced range shifts of three tick species in Europe

Sarah Cunze, Gustav Glock, Judith Kochmann, Sven Klimpel

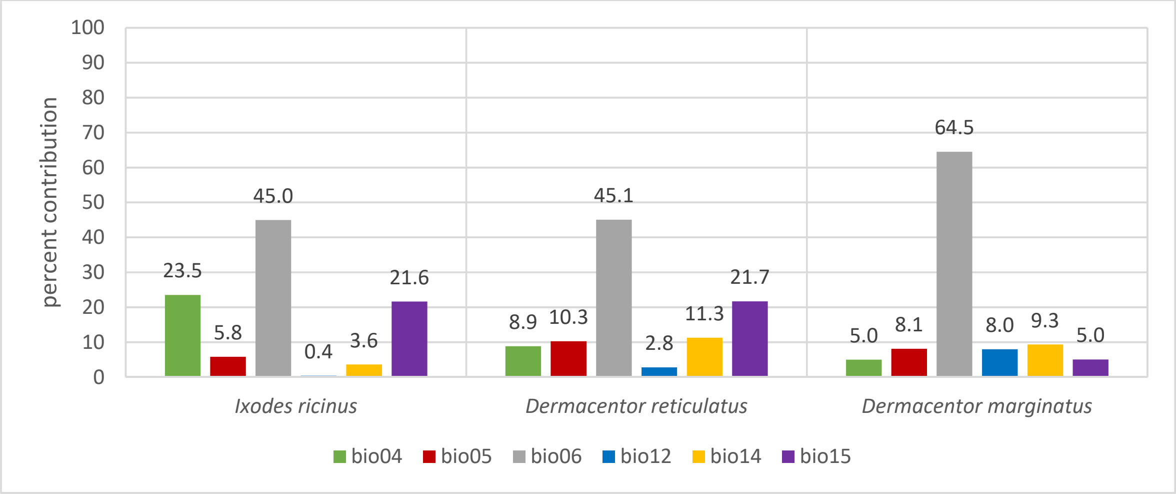

Figure S12: Estimates of relative contributions of the bioclimatic variables to the Maxent models for *Ixodes ricinus*, *Dermacentor reticulatus* and *Dermacentor marginatus*. Temperature seasonality (bio04), maximum temperature of warmest month (bio05), minimum temperature of coldest month (bio06), annual precipitation (bio12), precipitation of driest month (bio14) and precipitation seasonality (bio15).
